# Palladium-Catalyzed Acetoxylation of γ-Dehydro-aryl-himachalene: The Synthesis of a Novel Allylic Acetoxylated Sesquiterpene and a π-Allyl Palladium(II) Complex

**DOI:** 10.3390/molecules29215040

**Published:** 2024-10-25

**Authors:** Issam Louchachha, Abdelmajid Faris, Youssef Edder, Ali Hasnaoui, Anna Kozakiewicz-Piekarz, Abdelkarim Ait Mansour, Brahim Boualy, Rachid Salghi, Khalil Azzaoui, Rachid Sabbahi, Ashwag S. Alanazi, Mohamed Hefnawy, Belkheir Hammouti, Abdallah Karim, Mustapha Ait Ali

**Affiliations:** 1 Laboratory of Molecular Chemistry, Faculty of Sciences Semlalia, Cadi Ayyad University, B.P. 2390, Marrakech 40001, Morocco; louchachhaissam@gmail.com (I.L.); abdelmajid.faris@gmail.com (A.F.); youssef.edder@edu.uca.ac.ma (Y.E.); a.hasnaoui@uca.ac.ma (A.H.); karim@uca.ac.ma (A.K.); aitali@uca.ac.ma (M.A.A.); 2 Department of Chemistry, Faculty of Science, Chouaib Doukkali University, B.P. 299, El Jadida 24000, Morocco; 3 Department of Biomedical Chemistry and Polymers, Faculty of Chemistry, Nicolaus Copernicus University in Toruń, Gagarina 7, 87-100 Torun, Poland; akoza@chem.umk.pl; 4 Laboratory of Applied Chemistry and Environment, ENSA, University Ibn Zohr, P.O. Box 1136, Agadir 80000, Morocco; aitmansourabdelkarim8@gmail.com (A.A.M.); r.salghi@uiz.ac.ma (R.S.); 5 Multidisciplinary Research and Innovation Laboratory, Faculté Polydisciplinaire de Khouribga, Université Sultan Moulay Slimane, Khouribga 23000, Morocco; b.boualy@gmail.com; 6 Euromed Research Center, Euromed Polytechnic School, Euromed University of Fes, Eco-Campus, Fes Meknes Road, Fes 30030, Morocco; k.azzaoui@yahoo.com (K.A.); hammoutib@gmail.com (B.H.); 7 Laboratory of Organometallic, Molecular Materials and Environment, Faculty of Sciences, Sidi Mohammed Ben Abdellah University, Fez 30000, Morocco; 8 Research Team in Science and Technology, Higher School of Technology, University of Ibn Zohr, Laayoune 70000, Morocco; 9 Department of Pharmaceutical Sciences, College of Pharmacy, Princess Nourah bint Abdulrahman University, Riyadh 11671, Saudi Arabia; asalanzi@pnu.edu.sa; 10 Department of Pharmaceutical Chemistry, College of Pharmacy, King Saud University, Riyadh 11451, Saudi Arabia; mhefnawy@ksu.edu.sa

**Keywords:** sesquiterpenes, palladium-catalyzed acetoxylation, himachalenes, η^3^-allyl palladium complex, DFT calculations

## Abstract

Allylic oxygenated derivatives of himachalenes are highly valued molecules due to their potential applications in perfumery, cosmetics, and pharmaceuticals. Previous attempts at catalyzed allylic oxidation of himachalenes led to the formation of a very stable η^3^-allyl palladium complex, preventing any further reaction development. Herein, we present the first successful palladium-catalyzed synthesis of a novel allylic acetoxylated derivative of himachalenes. This reaction was achieved by incorporating an aromatic ring into the substrate structure. The resulting intermediate complex was isolated and characterized using nuclear magnetic resonance spectroscopy and X-ray crystallography. Density functional theory (DFT) calculations were performed to compare the reactivity of the newly synthesized complex with previously reported ones. The theoretical results confirm that the introduction of an aromatic ring enhances the reactivity of the η³-allyl palladium complex, thereby facilitating the desired transformation.

## 1. Introduction

Terpenes are undoubtedly important compounds with applications in a wide variety of domains, such as pharmaceuticals, cosmetics, perfumery, and synthetic chemistry [1,2]. These compounds are generally found in plant extracts and can be obtained by using different extraction and separation techniques [1,3]. Essential oils are one of these plant extracts that contain volatile compounds, mainly mono- and sesquiterpenes [4,5]. They present an excellent renewable feedstock for chemical synthesis as they are rich in natural olefins. Functionalized terpenes are generally more desirable because of their enhanced properties in comparison with their unfunctionalized analogues [6]. Hence, extensive studies have been dedicated to functionalizing olefinic terpenes [7,8,9,10].

Palladium-catalyzed allylic oxidation stands out as one of the most elegant and effective reactions. This reaction was first achieved via the re-oxidation of palladium using CuCl_2_ as a terminal oxidant (Wacker process) [11] and then by using a variety of oxidants including Cu(II) salts [12], hypervalent iodine [13], and most widely benzoquinone [14]. Most recently, using special nitrogen-containing ligands allowed molecular oxygen to be employed as the sole terminal oxidant [15,16,17]. Mechanistically, the reaction involves the formation of an η^3^-allyl palladium complex, which undergoes nucleophilic attack to give the desired products and a Pd(0) that reacts with the re-oxidant to regenerate the catalyst [18,19]. However, due to the structural complexity of terpenes, this method still cannot be generalized despite the extensive studies dedicated to the palladium-catalyzed oxidation of natural olefins [20,21,22,23,24,25].

**Figure 1:**
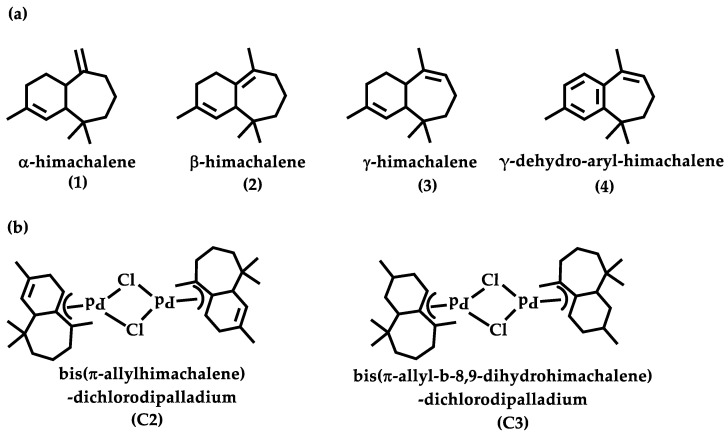
(**a**) Major constituents of cedar oil and (**b**) previously reported π-allyl palladium himachalene complexes.

Himachalenes (α- (**1**), β- (**2**), and γ-himachalene (**3**)) (Figure 1) are three sesquiterpene olefins found in cedar oils from *Cedrus atlantica*, *Cedrus deodara*, and *Cedrus libani*, with concentrations reaching up to 75% [26,27]. These compounds have undergone various chemical modifications to create novel molecules with enhanced olfactory and biological activities [28,29,30,31]. However, to our knowledge, no allylic oxygenated derivatives of these molecules have been reported in the literature, despite their promising properties [25,32,33].

Our research group’s previous attempts to use palladium-catalyzed allylic oxidation to obtain allylic oxygenated himachalene derivatives led to the formation of very stable π-allyl palladium complexes (**C2** and **C3**) (Figure 1) [34]. The high complex stability prevented any further evolution of the reaction toward the desired products. To circumvent these results, we introduced an aromatic ring into the himachalene structure by aromatization to obtain an activated alkene, γ-dehydro-aryl-himachalene (**4**) [35]. We reasoned that the introduction of an aromatic ring would destabilize the derived π-allyl complex, making it more susceptible to react in the presence of a nucleophile.

Herein, we report the first palladium-catalyzed allylic acetoxylation of himachalene derivatives with the isolation and characterization of a new π-allyl palladium γ-dehydro-aryl-himachalene complex, together with density functional theory (DFT) calculations and a comparative study between the newly synthesized complex (**C1**) and the previously reported complexes (**C2** and **C3**).

## 2. Results and Discussion

### 2.1. Acetoxylation of γ-Dehydro-aryl-himachalene

γ-Dehydro-aryl-himachalene (**4**) was obtained via the aromatization of a himachalene mixture (α- (**1**), β- (**2**) and γ-himachalene (**3**)), mediated by I_2_/DMSO, as we have previously reported [35]. The treatment of γ-dehydro-aryl-himachalene with Pd(OAc)_2_ in acetic acid and stoichiometric amounts of sodium acetate with benzoquinone as a re-oxidant was ineffective, as only 5% of the substrate reacted (Table 1), leading to the formation of trace amounts of the desired product (**6**). The substitution of benzoquinone with copper acetate slightly increased the substrate conversion to 11% and the allylic acetate selectivity to 14% (Table 1). 

Remarkably, using copper chloride as a re-oxidant resulted in the total conversion of the olefin, giving a gratifying 37% selectivity toward the desired product, with the formation of a chlorinated product (**5**) with 45% selectivity (Table 1). These results can be explained by the activating effect of CuCl_2_; Goncalves et al. [36] previously demonstrated that the role of copper chloride is not only limited to the oxidation of Pd(0) and the regeneration of the catalyst, but also includes facilitating the formation and reactivity of the key intermediate π-allyl complex by promoting the formation of organometallic species. Although the olefin was totally converted, the total yield of the desired allylic acetate (**6**) was moderate due to the formation of the expected undesired chlorinated derivative, product of an HCl addition on the double bond of γ-dehydro-aryl-himachalene (**4**), as CuCl_2_ is known to give this type of side product [37]. Varying the reaction parameters, such as increasing the temperature or using Li_2_PdCl_4_ as a catalyst with benzoquinone or copper acetate as re-oxidants, did not lead to any satisfying results, and the substrate reacted very poorly in these reaction conditions. 

**Figure 2:**
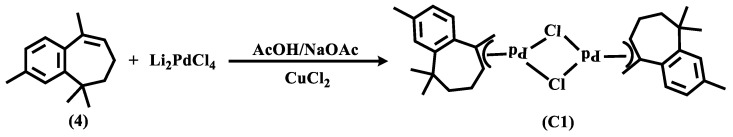
Synthesis of bis(π-allyl-γ-dehydro-aryl-himachalene)-dichlorodipalladium (**C1**).

The palladium-catalyzed allylic acetoxylation reaction involves the formation of a π-allyl palladium complex. The outcome of the reaction is strongly dependent on the reactivity of this complex. To better understand the reaction mechanism and the effect of aromatic rings on the reactivity of π-allyl palladium complexes, we synthesized and isolated the intermediate complex **C1** (Figure 2) using a previously reported method in the literature [38,39]. The complex was synthesized by reacting PdCl_2_ with γ-dehydro-aryl-himachalene in the presence of LiCl and CuCl_2_, using acetic acid as the solvent. It is noteworthy to mention that the presence of CuCl_2_ was mandatory to obtain the complex, as no formation of the latter was observed in the absence of copper chloride. This result correlates with the ones obtained above, as well as the findings previously reported by Goncalves et al. [36], confirming that copper chloride’s role is not only limited to the oxidation of Pd(0) but also intervenes in the formation of the π-allyl palladium dimer.

### 2.2. Crystal Structure Studies

**Figure 3:**
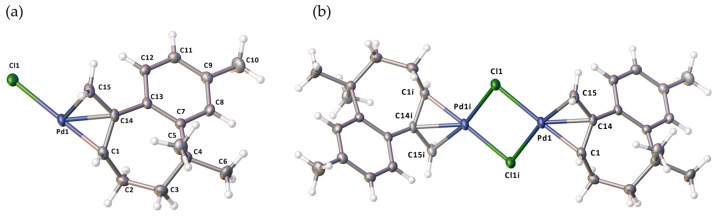
(**a**) The asymmetric unit and (**b**) the molecular structure of complex **C1**, with symmetry code i: −x + 1, −y + 1, −z + 2. The selected bond lengths (Å) and angles (°): Pd1-Cl1 2.4147(6), Pd1-Cl1i 2.4218(6), Pd1-C1 2.166(3), Pd1-C15 2.122(3), Pd1-C14 2.131(2), C1-Pd1-C15 68.99(11), C1-Pd1-C14 39.20(10), C15-Pd1-C14 38.95(10), C1-Pd1-Cl1 171.07(8), C15-Pd1-Cl1 104.38(8), C14-Pd1-Cl1 137.77(8), C1-Pd1-Cl1i 99.00(8), C15-Pd1-Cl1i 167.76(8), C14-Pd1-Cl1i 131.58(8), Cl1-Pd1-Cl1i 87.30(2), and Pd1-Cl1-Pd1i 92.70(2).

Structural studies indicated that the pale yellow crystals of complex **C1** crystallize in the monoclinic system in the P2_1_/c space group. This complex is a neutral and centrosymmetric binuclear Pd(II) complex, and the asymmetric part of the structure holds half of the molecule (Figure 3). The two halves of the dimer are related by the center of symmetry. The ligand binds in an ƞ^3^-ally manner to the metal ion via the C1, C14, and C15 atoms. The two metal atoms are bridged by two Cl- ions, forming a square ring Pd_2_Cl_2_, with the torsion angle of Pd1-Cl1-Pd1i-Cl1i [i = −x + 1, −y + 1, −z + 2] being 0.0°. A weak metal–metal interaction was observed, with a Pd…Pd distance of 3.500 Å. This distance is similar to the distance observed in these types of Pd(II) dimeric complexes (3.495 Å [34], 3.492 Å [40]). The bond lengths and angles in the metal coordination sphere (Figure 3) are not significantly different from those observed in similar complexes [34,39,40,41]. The geometry surrounding the palladium ion is four-coordinated, featuring a PdCl_2_C_2_ coordination environment. Based on the Addison parameter (τ = 0.147), this arrangement is classified as a distorted square planar. Notably, the C14 atom is positioned 0.700 Å above the best plane defined by the Cl_2_C_2_ arrangement.

**Figure 4:**
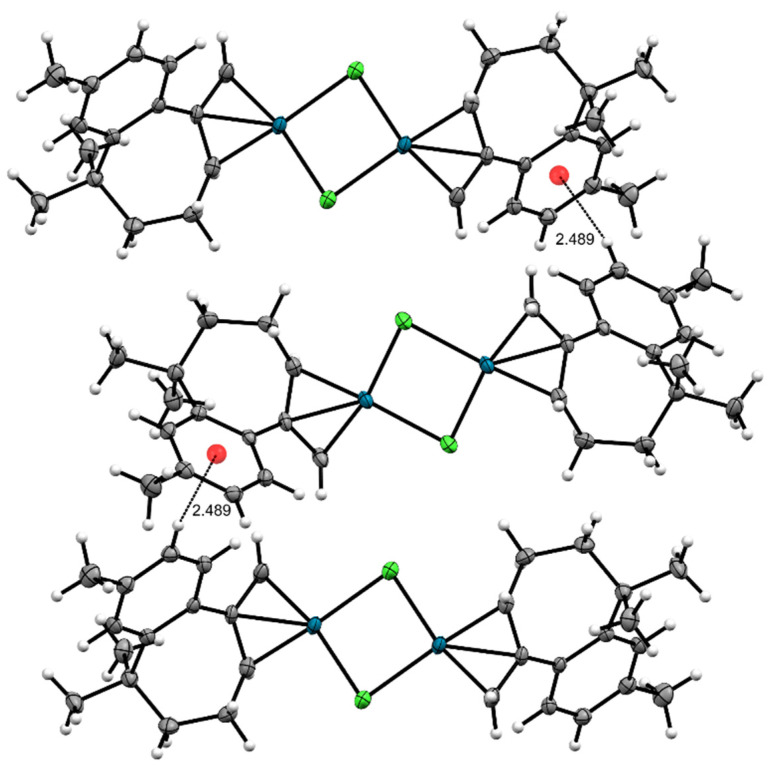
Crystal packing of complex **C1** showing C11-H11A…πC7--C13[x, ½ − y, ½ + z] interactions. Carbon atoms are marked in grey, hydrogen atoms in white, palladium atoms in blue and chlorine atoms in green.

Analysis of the crystal packing in **C1** revealed the presence of C11-H11A…πC7--C13[x, ½ − y, ½ + z] interactions with a distance of 2.489(3) Å (Figure 4).

### 2.3. Theoretical Results

To study the effect of the aromatic ring on the reactivity of the newly synthesized π-allyl palladium complex, it is imperative to examine the local and global reactivity of **C1** and **C2** to identify the most reactive complex. Analysis of the stability and reactivity of organic and inorganic molecules requires the use of density functional theory (DFT) [42]. This theoretical method was employed in the present study to identify which of the two complexes, **C1** or **C2**, is more reactive.

**Figure 5:**
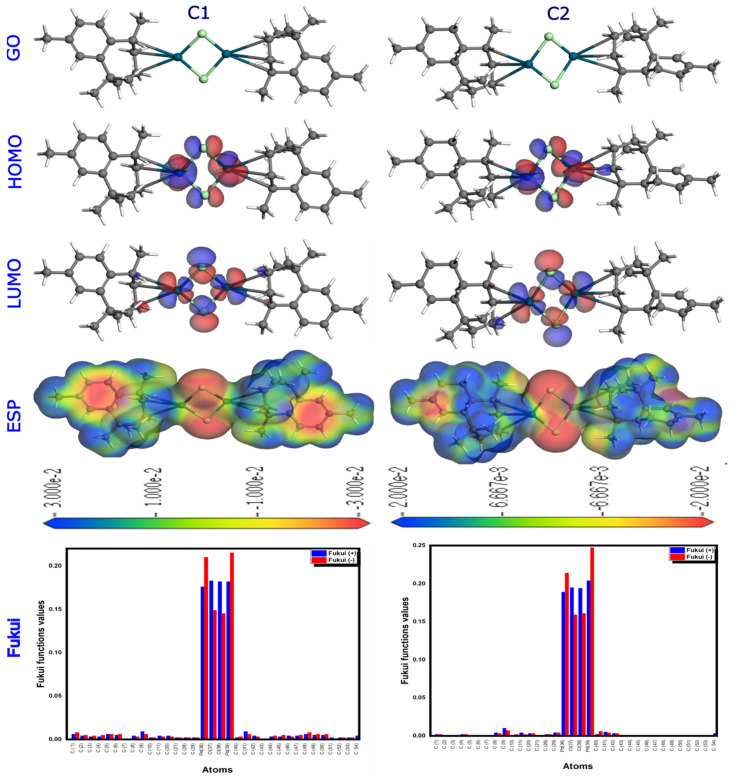
The geometry optimization (GO), highest occupied molecular orbital (HOMO), lowest unoccupied molecular orbital (LUMO), ESP isosurface, and Fukui indices of the complexes **C1** and **C2** derived by using the DFT/GGA tool.

Figure 5 illustrates the geometric optimization (GO), the most occupied molecular orbital (HOMO), the lowest unoccupied molecular orbital (LUMO), the ESP isosurface, and the Fukui indices of the two complexes, **C1** and **C2**, as obtained by the DFT/GGA approach. The palladium and chlorine atoms are at the center of the electron distribution, as shown by the HOMO and LUMO frontier orbitals, suggesting that both complexes are extremely reactive. In addition, Table 2 presents an overview of quantum characteristics, such as the HOMO and LUMO energies, electronegativity, and energy gap (ΔEgap). Our data indicate that the **C1** complex has a smaller energy gap (ΔEgap) than the **C2** complex, which also has a smaller energy gap than the previously reported **C3** complex by Faris et al. [34] ((ΔEgap = 4.72 eV) ΔEgap (**C1**) < ΔEgap (**C2**) < ΔEgap (**C3**)), indicating that the presence of aromatic rings that increase the reactivity of organic molecules through the conjugation effect makes the **C1** complex more susceptible to react with nucleophiles [43]. According to the literature, electrostatic potential (ESP) isosurfaces based on the total electron density surface have been used to study hydrogen bonding interactions and biological recognition. They also provide an intuitive representation of molecule size and shape, electrostatic potential distribution, chemically active sites, and atomic reactivity [44]. The ESP surface was calculated using Material Studio software (Figure 5). The electron-rich zone (negative ESP region), the electron-poor zone (positive ESP region), and the neutral zone are represented by the red, blue, and green colors of the ESP surface, respectively [44]. The most negative (red) potential area of the **C1** complex is around the aromatic (phenyl) ring, while the most positive (blue) region is near the carbon atoms (CH_3_), but the **C2** complex carries only the bulk of it. According to the ESP isosurfaces, the **C1** complex contains more nucleophilic attack sites (blue regions on the methyl) and electrophilic attack sites (red regions on the phenyl ring) than the **C2** complex. In addition, this complex has more electrophilic and nucleophilic sites than complex **C2**, according to the Fukui indices shown in Figure 5. To sum up, the synthesis results are consistently supported by the combined data from the geometric optimization, molecular orbital analysis, ESP isosurfaces, and Fukui indices, which show that complex **C1** is more reactive than complex **C2**.

## 3. Materials and Methods

### 3.1. Materials

All chemicals and solvents were purchased from Sigma Aldrich and used without any further purification. ^1^H and ^13^C NMR spectra were recorded on a Bruker Avance 300 spectrometer (Bruker Biospin, Billercia, MA, USA), operating at 300 MHz for 1H NMR and 75 MHz for 13C NMR with tetramethylsilane as the internal reference. The chemical shifts (δ) were measured in ppm. Samples from the reaction mixture were monitored by Shimadzu gas chromatography (GC) with a flame ionization detector using nitrogen as a carrier gas. The GC parameters for the capillary column BP (25 m  ×  0.25 mm, SGE) are as follows: injector 250 °C; detector 250 °C; oven 70 °C for 5 min, then 3 min until 250 °C for 30 min; column pressure 20 kPa; column flow 6.3 mL/min; linear velocity 53.1 cm/s; total flow 138 mL/min. The products were confirmed by injecting the reaction mixture on an ISQ LT single quadrupole mass spectrometer (Thermo Scientific, Waltham, MA, USA) in positive EI mode using a mass scan range of 50 to 400 Da.

### 3.2. X-Ray Crystallographic Studies of Bis(π-allyl-γ-dehydro-aryl-himachalene)-dichlorodipalladium (***C1***)

The X-ray data for the yellow plate crystal of complex **C1** were collected at 100(2) K with a Rigaku XtaLAB Synergy-S diffractometer using CuKα radiation. The obtained data set was processed with CrysAlisPro 1.171.38.43 software [45]. The structure was solved by using direct methods and refined with the full-matrix least-squares method on F2 with the use of SHELX2014 program packages [46]. A summary of the crystal data and refinement details is given in Table 3. The structural data have been deposited at the Cambridge Crystallographic Data Center (www.ccdc.cam.ac.uk/data_request/cif, (accessed on 20 September 2024) CCDC No. 2368621).

### 3.3. Theoretical Calculations

To carry out theoretical calculations, the DFT method was used with “Material Studio” 6.0 software. As described in the Dmol3 technique, the generalized gradient approximation (GGA) and the digital double basis set with polarization (DNP) were used to optimize the molecular structures. Equations (1)–(7) were used to determine several quantum chemical parameters, such as the HOMO and LUMO energies, energy gap (ΔEgap), electron affinity (EA), ionization potential (IP), electronegativity (X), number of transported electrons (ΔNmax), and dipole moment properties (μ) [47]. To guarantee excellent results, the parameterization of the Dmol3 code was consistent with our theoretical calculations. In addition, Fukui function indices were evaluated using Equations (8) and (9) of the Hirshfeld population analysis, and the aqueous phase was simulated using the COSMO solvation model [48]. In addition, frontier molecular orbitals (FMOs) were used to assess local and global reactivities. By cross-checking the fine-grained fits with Dmol3 code parameters, the theoretical results were found to be consistent. VMD and Multiwfn were used to visualize the reduced density gradient (RDG) and the non-covalent interaction (NCI) structures.

(1) $${∆E}_{gap}=E_{LUMO}-E_{HOMO}=IP-EA$$

(2) $$IP={-E}_{HOMO}$$

(3) $$EA=-E_{LUMO}$$

(4) $$\chi_{inh}=\frac{IP+EA}{2}$$

(5) $$\eta_{inh}=\frac{{∆E}_{gap}}{2}$$

(6) $$\mu =\frac{E_{LUMO}+E_{HOMO}}{2}$$

(7) $${\Delta N}_{max}=\frac{-\mu }{\eta_{inh}}$$

Fk+ = qk (N + 1) − qk (N) (8)

Fk− = qk (N) − qk (N − 1)(9)

The electron densities on atom k corresponding to N + 1, (N − 1), and (N − 1) are designated by the symbols qk (N + 1), qk (N), and qk (N − 1). 

### 3.4. Acetoxylation Reaction of γ-Dehydro-aryl-himachalene

In a three-necked, round-bottomed flask equipped with a magnetic stir bar and a condenser, 10 mL of acetic acid and 213.4 mg (2.6 mmol) of sodium acetate were introduced and stirred till total dissolution of the base. Then, 22.44 mg (0.1 mmol) of Pd(OAc)_2_ and 268.9 mg (2 mmol) of CuCl_2_ were added and stirred at 60 °C till total dissolution (around 15 min). Finally, 200 mg (1 mmol) of γ-dehydro-aryl-himachalene was added, and the reaction mixture was stirred for 48 h. The reaction was then quenched with a saturated solution of NaHCO_3_ and extracted three times with 20 mL of diethyl ether. Organic layers were gathered and washed 3 times with 20 mL of deionized water, dried over MgSO_4_, and filtered. The solvent was removed in vacuo. The crude mixture was separated using silica gel column chromatography and eluted with hexane/ethyl acetate (98/2). 

5-Chloro-2,5,9,9-tetramethyl-6,7,8,9-tetrahydro-5H-benzo[7]annulene (**5**)

Yield: 40% (95 mg), colorless liquid, NMR ^1^H (300 MHz, CDCl_3_, δ ppm): 7.16-6.96 (3H, m, H-Ar), 2.28 (3H, s, CH_3_-Ar), 2.24-2.16 (2H, 3, CH_2_), 2.07 (3H, s, CH_3_), 2.03-1.99 (2H, m, CH_2_), 1.23 (6H, s, 2xCH_3_), 1.19-1.17 (2H, m, CH_2_).

NMR ^13^C (75 MHz, CDCl_3_, δ ppm): 146.28; 142.03; 136.37; 128.10; 126.54; 126.43; 70.28; 47.69; 37.82; 35.20; 31.59; 29.73; 21.55; 20.33.

Molecular weight: *m*/*z* = 236.1.

(Supplementary Materials)

(3,5,5-Trimethyl-6,7-dihydro-5H-benzo[7]annulen-9-yl)methyl acetate (**6**)

Yield: 31% (80 mg), yellow liquid, NMR ^1^H (300 MHz, CDCl_3_, δ ppm): 7.31-7.29 (2H, m, H-Ar), 7.12-7.09 (1H, m, H-Ar), 6.27-6.23 (1H, m, CH=C), 5.01 (2H, s, CH_2_), 2.42 (3H, s, CH_3_), 2.25-2.14 (4H, m, 2xCH_2_), 2.08 (3H, s, CH_3_-Ar), 1.40 (6H, m, 2xCH_3_).

NMR ^13^C (75 MHz, CDCl_3_, δ ppm): 170.86; 140,29; 136,51; 136,14; 134.15; 131.27; 127.56; 126.70; 126.50; 67.94; 47.54; 38.09; 31.30; 26.21; 21.60; 21.03.

Molecular weight: *m*/*z* = 258.2

(Supplementary Materials)

### 3.5. Synthesis of Bis(π-allyl-γ-dehydro-aryl-himachalene)-dichlorodipalladium (***C1***)

In a three-necked, round-bottomed flask equipped with a magnetic stir bar and a condenser, 10 mL of acetic acid and 114.84 mg (1.4 mmol) of sodium acetate were introduced and stirred till the total dissolution of the base. Then, 100 mg (0.56 mmol) of PdCl_2_ and 29.67 mg (1.38 mmol) of lithium chloride were added, and the reaction mixture was stirred at 85 °C for 30 min. Then, the reaction mixture was cooled to 60 °C, and 227 mg (1.12 mmol) of γ-dehydro-aryl-himachalene was introduced. The reaction mixture was stirred for 12 h. At the end of the reaction, monitored by TLC, the reaction mixture was cooled to room temperature and extracted with CHCl_3_ (3 × 20 mL); the organic layers were gathered and washed with a saturated solution of NaHCO_3_ (2 × 30 mL), dried over MgSO_4_, and filtered. The solvent was evaporated under reduced pressure. The complex was purified using silica gel column chromatography and eluted with hexane and hexane/ethyl acetate (95/5). The recrystallization of the product was performed by slow evaporation from a hexane solution.

Yield: 56%, pale yellow crystals, NMR ^1^H (300 MHz, CDCl_3_, δ ppm): 7.67-7.64 (1H, m, H-Ar), 7.32-7.23 (1H, m, H-allyl), 7.02-7.00 (2H, m, H-Ar), 5.44-5.40 (1H, m, H-allyl), 4.60-4.55 (1H, m, H-allyl), 2.25-2.16 (3H, M, CH3), 1.96-1.88 (3H, m, CH3), 1.83-1.75 (2H, m, CH2), 1.32-1.27 (2H, m, CH2), 1.22-1.16 (3H, M, CH3).

NMR ^13^C (75 MHz, CDCl_3_, δ ppm): 167.00; 144.11; 136.92; 132.71; 131.23; 127.65; 126.23; 105.42; 88.33; 48.78; 36.83; 31.41; 31.19; 27.45; 25.57. (Supplementary Materials)

## 4. Conclusions

In summary, through the introduction of an aromatic ring to the structure of himachalenes and the synthesis of γ-dehydro-aryl-himachalene, we have successfully obtained a novel allylic acetoxylated derivative of himachalene via the palladium-catalyzed acetoxylation of himachalenes. The intermediate π-allyl palladium complex was also isolated and characterized using NMR spectroscopy and X-ray crystallography. DFT calculations of the reactivity of the novel synthesized complex, as well as the ones previously reported, confirmed the hypothesis that the introduction of an aromatic ring enhances the reactivity of η^3^-allyl palladium complexes, thereby facilitating the desired transformation.

## Figures and Tables

**Table 1:** Optimization of the acetoxylation reaction of γ-dehydro-aryl-himachalene.

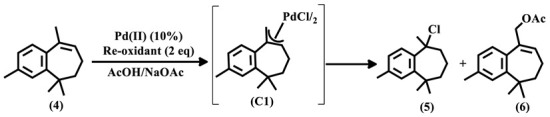								
Entry	[Pd]	Re-Oxidant	Temperature (°C)	Time (h)	Conversion (%) ^1^	Product (5) (%) ^2^	Product (6) (%) ^2^	Other ^2^ (%)
1	Pd(OAc)_2_	BQ	60	96	5	-	tr ^3^	Tr ^3^
2	Pd(OAc)_2_	Cu(OAc)_2_	60	96	11	-	14	86
3	Pd(OAc)_2_	CuCl_2_	60	f48	100	46	37	17
4	Pd(OAc)_2_	Cu(OAc)_2_	80	96	24	-	12	88
5	Pd(OAc)_2_	BQ	80	96	40	-	9	91
6	Li_2_PdCl_4_	CuCl_2_	60	72	100	45	35	20
7	-	CuCl_2_	60	72	100	85	-	15

Reaction conditions: γ-dehydro-aryl-himachalene (1 mmol), NaOAc (2.6 mmol), [Pd] (0.1 mmol), CuCl_2_ (2 mmol), and AcOH (10 mL). ^1^ determined by GC. ^2^ calculated based on reacted substrate. ^3^ trace amounts.

**Table 2:** The chemical quantum parameters of the **C1** and **C2** complexes, established using the DFT/GGA theoretical model.

Complex	E_HOMO_	E_LUMO_	IP	EA	ΔE_gap_	ᵡinh	Ƞinh	ΔNinh
**C1**	−5.396	−5.044	5.396	5.044	0.352	5.22	0.176	29.659
**C2**	−5.116	−4.688	5.116	4.688	0.428	4.902	0.214	22.906

All quantum parameters are presented in electronvolts (eV).

**Table 3:** Some data from the single-crystal X-ray analysis of complex **C1**.

	C1
Empirical formula	C_30_H_38_Cl_2_Pd_2_
Mr/g mol^−1^	682.30
Crystal system	Monoclinic
Space group	P2_1_/c
Crystal size (mm)	0.17 × 0.03 × 0.03
a (Å)	16.6259(3)
b (Å)	12.5859(2)
c (Å)	6.63845(11)
α (°)	90
β (°)	95.6534(16)
γ (°)	90
Z	2
Cell volume (Å^3^)	1382.35(4)
Density (Mg m^−3^)	1.639
µ (mm^−1^)	12.385
F(000)	688
θ Range (°)	2.7–79.4
T_min_, T_max_	0.552, 1.000
Measured reflections	4443
Independent reflections	4443
R_int_	0.043
h	−21→21
k	−15→15
l	−7→8
S	1.096
Parameters, restraints	158, 0
R1[F^2^ > 2σ(F^2^)]	0.025
wR(F^2^)	0.07
Δρmin; Δρmax (e Å^−3^)	−0.54, 0.93

## Data Availability

The data presented in this study are available upon request from the corresponding author.

## Supplementary Materials

The following supporting information can be downloaded at: https://www.mdpi.com/article/10.3390/molecules29215040/s1, Figure S1. ^1^H NMR Spectrum of **5**. Figure S2. ^13^C NMR Spectrum of **5**. Figure S3. MS Spectrum of **5**. Figure S4. ^1^H NMR Spectrum of **6**. Figure S5. ^13^C NMR Spectrum of **6**. Figure S6. MS Spectrum of **6**. Figure S7. ^1^H NMR Spectrum of **C1**. Figure S8. ^13^C NMR Spectrum of **C1**.

## References

[1] Silvestre A.J.D., Gandini A. (2008). Terpenes: Major Sources, Properties and Applications. Monomers, Polymers and Composites from Renewable Resources.

[2] Cox-Georgian D., Ramadoss N., Dona C., Basu C. (2019). Therapeutic and Medicinal Uses of Terpenes. Medicinal Plants.

[3] Jiang Z., Kempinski C., Chappell J. (2016). Extraction and Analysis of Terpenes/Terpenoids. Curr. Protoc. Plant Biol..

[4] Andoğan B.C., Baydar H., Kaya S., Demirci M., Özbaşar D., Mumcu E. (2002). Antimicrobial Activity and Chemical Composition of Some Essential Oils. Arch. Pharm. Res..

[5] Eslahi H., Fahimi N., Sardarian A.R. (2017). Chemical Composition of Essential Oils. Essential Oils in Food Processing.

[6] Böttger A., Vothknecht U., Bolle C., Wolf A. (2018). Terpenes and Terpenoids.

[7] Schwab W., Fuchs C., Huang F. (2013). Transformation of Terpenes into Fine Chemicals. Eur. J. Lipid Sci. Technol..

[8] Ravasio N., Zaccheria F., Guidotti M., Psaro R. (2004). Mono- and Bifunctional Heterogeneous Catalytic Transformation of Terpenes and Terpenoids. Top. Catal..

[9] Swift K.A.D. (2004). Catalytic Transformations of the Major Terpene Feedstocks. Top. Catal..

[10] Monteiro J.L.F., Veloso C.O. (2004). Catalytic Conversion of Terpenes into Fine Chemicals. Top. Catal..

[11] Michel B.W., Steffens L.D., Sigman M.S. (2014). Wacker Oxidation, The. Organic Reactions.

[12] Hosokawa T., Murahashi S. (1990). New Aspects of Oxypalladation of Alkenes. Acc. Chem. Res..

[13] Pilarski L.T., Selander N., Böse D., Szabó K.J. (2009). Catalytic Allylic C−H Acetoxylation and Benzoyloxylation via Suggested (η^3^-Allyl)Palladium(IV) Intermediates. Org. Lett..

[14] Popp B.V., Stahl S.S. (2007). Palladium-Catalyzed Oxidation Reactions: Comparison of Benzoquinone and Molecular Oxygen as Stoichiometric Oxidants. Organometallic Oxidation Catalysis.

[15] Campbell A.N., Stahl S.S. (2012). Overcoming the “Oxidant Problem”: Strategies to Use O_2_ as the Oxidant in Organometallic C–H Oxidation Reactions Catalyzed by Pd (and Cu). Acc. Chem. Res..

[16] Campbell A.N., White P.B., Guzei I.A., Stahl S.S. (2010). Allylic C–H Acetoxylation with a 4,5-Diazafluorenone-Ligated Palladium Catalyst: A Ligand-Based Strategy To Achieve Aerobic Catalytic Turnover. J. Am. Chem. Soc..

[17] Wang D., Weinstein A.B., White P.B., Stahl S.S. (2018). Ligand-Promoted Palladium-Catalyzed Aerobic Oxidation Reactions. Chem. Rev..

[18] Fernandes R.A., Nallasivam J.L. (2019). Catalytic Allylic Functionalization via π-Allyl Palladium Chemistry. Org. Biomol. Chem..

[19] Liron F., Oble J., Lorion M.M., Poli G. (2014). Direct Allylic Functionalization Through Pd-Catalyzed C–H Activation. Eur. J. Org. Chem..

[20] Gonçalves J.A., Gusevskaya E.V. (2004). Palladium Catalyzed Oxidation of Monoterpenes: Multistep Electron Transfer Catalytic Systems Pd(OAc)_2_/Benzoquinone/M(OAc)_2_ (M=Cu, Co or Mn) for the Allylic Oxidation of Limonene with Dioxygen. Appl. Catal. A Gen..

[21] Gonçalves J.A., Howarth O.W., Gusevskaya E.V. (2002). Palladium Catalyzed Oxidation of Monoterpenes: Novel Oxidation of Myrcene with Dioxygen. J. Mol. Catal. A Chem..

[22] El Firdoussi L., Baqqa A., Allaoud S., Ait Allal B., Karim A., Castanet Y., Mortreux A. (1998). Selective Palladium-Catalysed Functionalization of Limonene: Synthetic and Mechanistic Aspects. J. Mol. Catal. A Chem..

[23] Speziali M.G., Costa V.V., Robles-Dutenhefner P.A., Gusevskaya E.V. (2009). Aerobic Palladium(II)/Copper(II)-Catalyzed Oxidation of Olefins Under Chloride-Free Nonacidic Conditions. Organometallics.

[24] Speziali M.G., Robles-Dutenhefner P.A., Gusevskaya E.V. (2007). Palladium-Catalyzed Oxidation of Monoterpenes: Novel Aerobic Pd(II)/Cu(II)-Catalyzed Oxidation of Linalool Under Chloride-Free Nonacidic Conditions. Organometallics.

[25] Venancio A.N., Menini L., Maronde D.N., Gusevskaya E.V., Parreira L.A. (2021). Palladium Catalyzed Oxidation of Biorenewable β-Citronellol and Geraniol for the Synthesis of Polyfunctionalized Fragrances. Mol. Catal..

[26] Chalchat J.C., Garry R.P., Miehet A., Benjilali B. (1994). Essential Oil Components in Sawdust of *Cedrus atlantica* from Morocco. J. Essent. Oil Res..

[27] Aberchane M., Fechtal M., Chaouch A. (2004). Analysis of Moroccan Atlas Cedarwood Oil (*Cedrus atlantica* Manetti). J. Essent. Oil Res..

[28] Ait Lahcen I., Edder Y., Louchachha I., Faris A., Boualy B., Karim A. (2024). Synthesis of Novel Amide Derivatives of the Sesquiterpene Aryl-Himachalene. Org. Prep. Proced. Int..

[29] Faris A., Edder Y., Louchachha I., Lahcen I.A., Azzaoui K., Hammouti B., Merzouki M., Challioui A., Boualy B., Karim A. (2023). From Himachalenes to Trans-Himachalol: Unveiling Bioactivity through Hemisynthesis and Molecular Docking Analysis. Sci. Rep..

[30] Yamini Y., Anand P., Bhardwaj V.K., Kumar A., Purohit R., Das P., Padwad Y. (2023). Novel Pyrrolone-Fused Benzosuberene MK2 Inhibitors: Synthesis, Pharmacophore Modelling, Molecular Docking, and Anti-Cancer Efficacy Evaluation in HNSCC Cells. J. Biomol. Struct. Dyn..

[31] Chaudhary A., Das P., Mishra A., Kaur P., Singh B., Goel R.K. (2012). Naturally Occurring Himachalenes to Benzocycloheptene Amino Vinyl Bromide Derivatives: As Antidepressant Molecules. Mol. Divers..

[32] Gusevskaya E.V., Jiménez-Pinto J., Börner A. (2014). Hydroformylation in the Realm of Scents. ChemCatChem.

[33] dos Santos Costa M., de Camargo Faria A., Gusevskaya E.V. (2019). New Scents from Bio-Renewable Cis-Jasmone by Aerobic Palladium Catalyzed Oxidations. Appl. Catal. A Gen..

[34] Faris A., Edder Y., Hdoufane I., Ait Lahcen I., Saadi M., EL Ammari L., Berraho M., Cherqaoui D., Boualy B., Karim A. (2023). Syntheses, Characterization and DFT Studies of Two New (π-Allyl) Palladium(II) Complexes of β-8,9-Dihydrohimachalene. J. Coord. Chem..

[35] Isam L., Youssef E., Abdelmajid F., Boualy B., Ali M.A., Karim A. (2024). Iodine-Mediated Aromatization of Himachalenes: Synthesis of Dehydro-7,8-Arylhimachalene. Lett. Org. Chem..

[36] Gonçalves J.A., da Silva M.J., Piló-Veloso D., Howarth O.W., Gusevskaya E.V. (2005). Palladium Catalyzed Oxidation of Monoterpenes: NMR Study of Palladium(II)–Monoterpene Interactions. J. Organomet. Chem..

[37] Keith J.A., Nielsen R.J., Oxgaard J., Goddard W.A. (2007). Unraveling the Wacker Oxidation Mechanisms. J. Am. Chem. Soc..

[38] Chiaroni A., Riche C., El Firdoussi L., Benharref A., Karim A. (1993). Structure Du Bis(π-Allylhimachalène)-α,α-Dichlorodipalladium. Acta Crystallogr. Sect. C.

[39] El Firdoussi L., Allaoud S., Karim A., Barrero A.F., Quirós M., Castanet Y., Mortreux A. (1997). Bis(π-Allyl-6,7-Dihydrohimachalene)-α,α-Dichlorodipalladium. Acta Crystallogr. Sect. C.

[40] Sui-Seng C., Enright G.D., Zargarian D. (2004). New Routes to η^1^-and (η^3^↔η^5^)-Indenylpalladium Complexes: Synthesis, Characterization, and Reactivities. Organometallics.

[41] Fernandes R.A., Nallasivam J.L. (2012). Enantioselective Allylation of Imines Catalyzed by Newly Developed (−)-β-Pinene-Based π-Allylpalladium Catalyst: An Efficient Synthesis of (R)-α-Propylpiperonylamine and (R)-Pipecolic Acid. Org. Biomol. Chem..

[42] Ahmad S., Nadeem S., Anwar A., Hameed A., Tirmizi S.A., Zierkiewicz W., Abbas A., Isab A.A., Alotaibi M.A. (2017). Synthesis, Characterization, DFT Calculations and Antibacterial Activity of Palladium(II) Cyanide Complexes with Thioamides. J. Mol. Struct..

[43] Fırıncı R., Günay M.E., Özdemir N., Dinçer M. (2017). Synthesis, Structural, Spectroscopic and DFT Study on a Palladium(II)-N-Heterocyclic Carbene Complex. J. Mol. Struct..

[44] Chai Y.-M., Zhang H.-B., Zhang X.-Y., Chai L.-Q. (2022). X-Ray Structures, Spectroscopic, Antimicrobial Activity, ESP/HSA and TD/DFT Calculations of Bi(III) Complex Containing Imidazole Ring. J. Mol. Struct..

[45] Oxford Diffraction (2015). CrysAlisPro.

[46] Sheldrick G.M. (2015). *SHELXT*—Integrated Space-Group and Crystal-Structure Determination. Acta Crystallogr. A Found. Adv..

[47] Ait Mansour A., Lgaz H., Elmoutaouakil Ala Allah A., Ramli Y., Messali M., Lee H., Bazzi L., Salghi R., Hammouti B. (2024). Comprehensive Analysis of a Thiazole-Substituted Corrosion Inhibitor’s Impact on N80 Carbon Steel in Acidic Conditions: Integrating Computational Predictions with Experimental Verifications. Mater. Chem. Phys..

[48] Ait Mansour A., Lgaz H., Elmoutaouakil Ala Allah A., Jang J., Messali M., Bazzi L., Lee H., Ramli Y., Salghi R. (2024). Indolin-2-One Derivatives as Corrosion Inhibitors: Structural Insights and Evaluation through Experimental and Computational Techniques. J. Mol. Struct..

