# Supplementary material for: Palladium-Catalyzed Acetoxylation of γ-Dehydro-aryl-himachalene: The Synthesis of a Novel Allylic Acetoxylated Sesquiterpene and a π-Allyl Palladium(II) Complex
Source: Molecules. 2024 Oct 25;29(21):5040. doi: 10.3390/molecules29215040 (PMC11547928; doi:10.3390/molecules29215040)
Supplement: Supplementary file 1 [file molecules-29-05040-s001.zip › molecules-3245287-supplementary.pdf]

Palladium-Catalyzed Acetoxylation of γ-Dehydro-aryl-himachalene: The Synthesis of a Novel Allylic Acetoxylated Sesquiterpene and a π-Allyl Palladium(II) Complex

Issam Louchachha [1], Abdelmajid Faris [1], Youssef Edder [1,2], Ali Hasnaoui [1], Anna Kozakiewicz-Piekarz [3], Abdelkarim Ait Mansour [4], Brahim Boualy [5], Rachid Salghi [4,6], Khalil Azzaoui [6,7], Rachid Sabbahi [6,8,*], Ashwag S. Alanazi [9], Mohamed Hefnawy [10], Belkheir Hammouti [6], Abdallah Karim [1] and Mustapha Ait Ali [1]

[1] Laboratory of Molecular Chemistry, Faculty of Sciences Semlalia, Cadi Ayyad University, B.P. 2390, Marrakech 40001, Morocco; louchachhaissam@gmail.com (I.L.); abdelmajid.faris@gmail.com (A.F.); youssef.edder@edu.uca.ac.ma (Y.E.); a.hasnaoui@uca.ac.ma (A.H.); karim@uca.ac.ma (A.K.); aitali@uca.ac.ma (M.A.A.)

[2] Department of Chemistry, Faculty of Science, Chouaib Doukkali University, B.P. 299, El Jadida 24000, Morocco

[3] Department of Biomedical Chemistry and Polymers, Faculty of Chemistry, Nicolaus Copernicus University in Toruń, Gagarina 7, 87-100 Torun, Poland; akoza@chem.umk.pl

[4] Laboratory of Applied Chemistry and Environment, ENSA, University Ibn Zohr, P.O. Box 1136, Agadir 80000, Morocco; aitmansourabdelkarim8@gmail.com (A.A.M.); r.salghi@uiz.ac.ma (R.S.)

[5] Multidisciplinary Research and Innovation Laboratory, Faculté Polydisciplinaire de Khouribga, Université Sultan Moulay Slimane, Khouribga 23000, Morocco; b.boualy@gmail.com

[6] Euromed Research Center, Euromed Polytechnic School, Euromed University of Fes, Eco-Campus, Fes Meknes Road, Fes 30030, Morocco; k.azzaoui@yahoo.com (K.A.); hammoutib@gmail.com (B.H.)

[7] Laboratory of Organometallic, Molecular Materials and Environment, Faculty of Sciences, Sidi Mohammed Ben Abdellah University, Fez 30000, Morocco

[8] Research Team in Science and Technology, Higher School of Technology, University of Ibn Zohr, Laayoune 70000, Morocco

[9] Department of Pharmaceutical Sciences, College of Pharmacy, Princess Nourah bint Abdulrahman University, Riyadh 11671, Saudi Arabia; asalanzi@pnu.edu.sa

[10] Department of Pharmaceutical Chemistry, College of Pharmacy, King Saud University, Riyadh 11451, Saudi Arabia; mhefnawy@ksu.edu.sa

* Correspondence: r.sabbahi@uiz.ac.ma

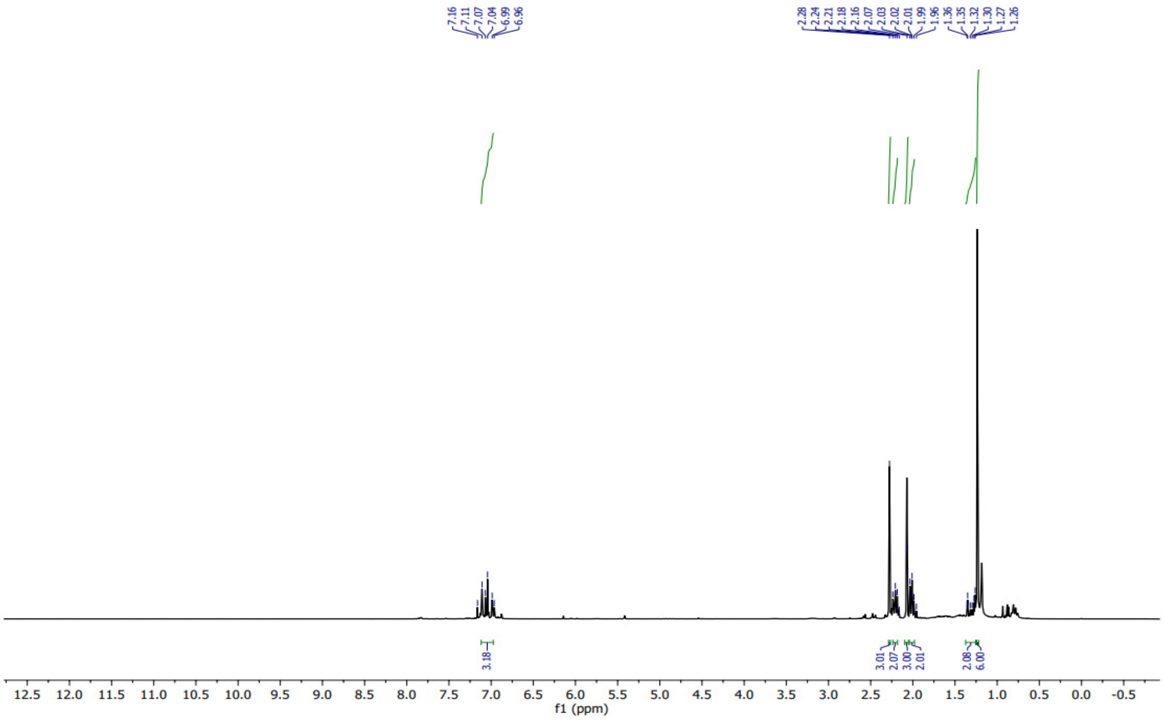

Figure S1. ^{1}H NMR Spectrum of **5**

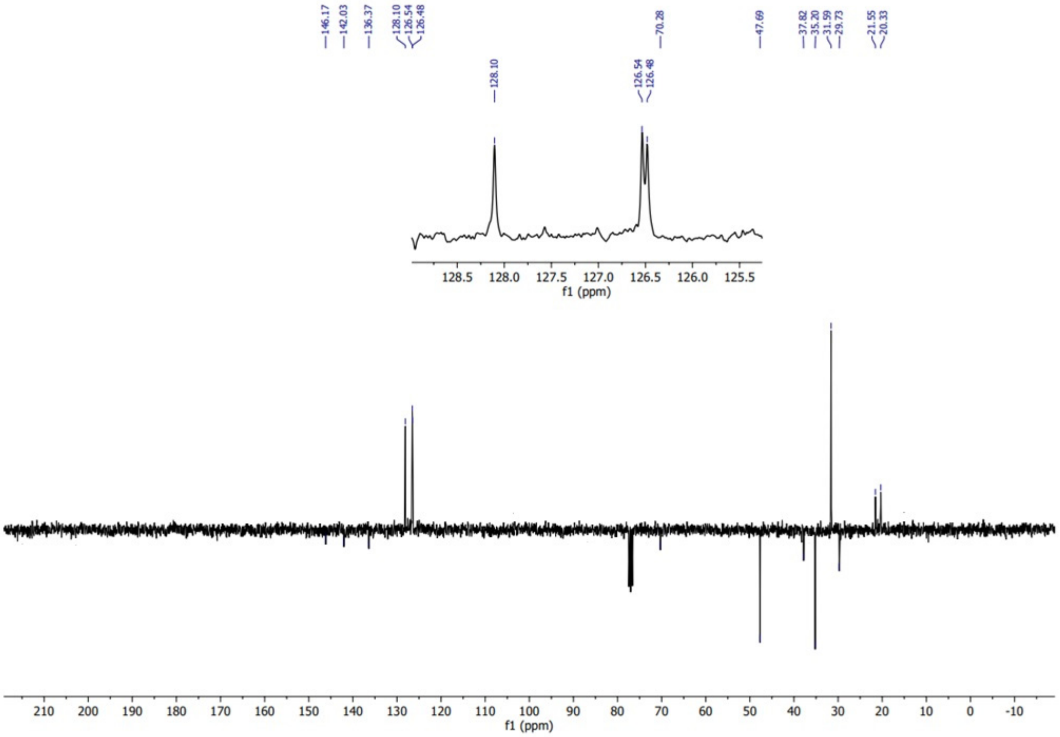

Figure S2. ^{13}C NMR Spectrum of **5**

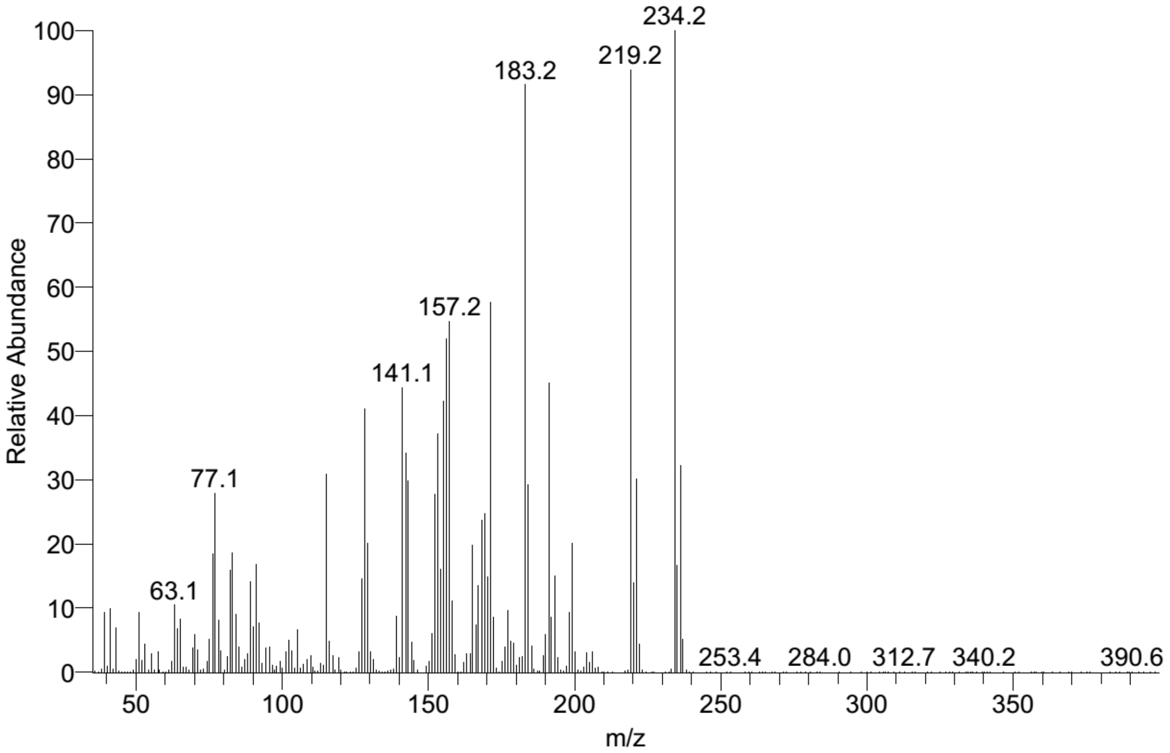

Figure S3. MS Spectrum of **5**

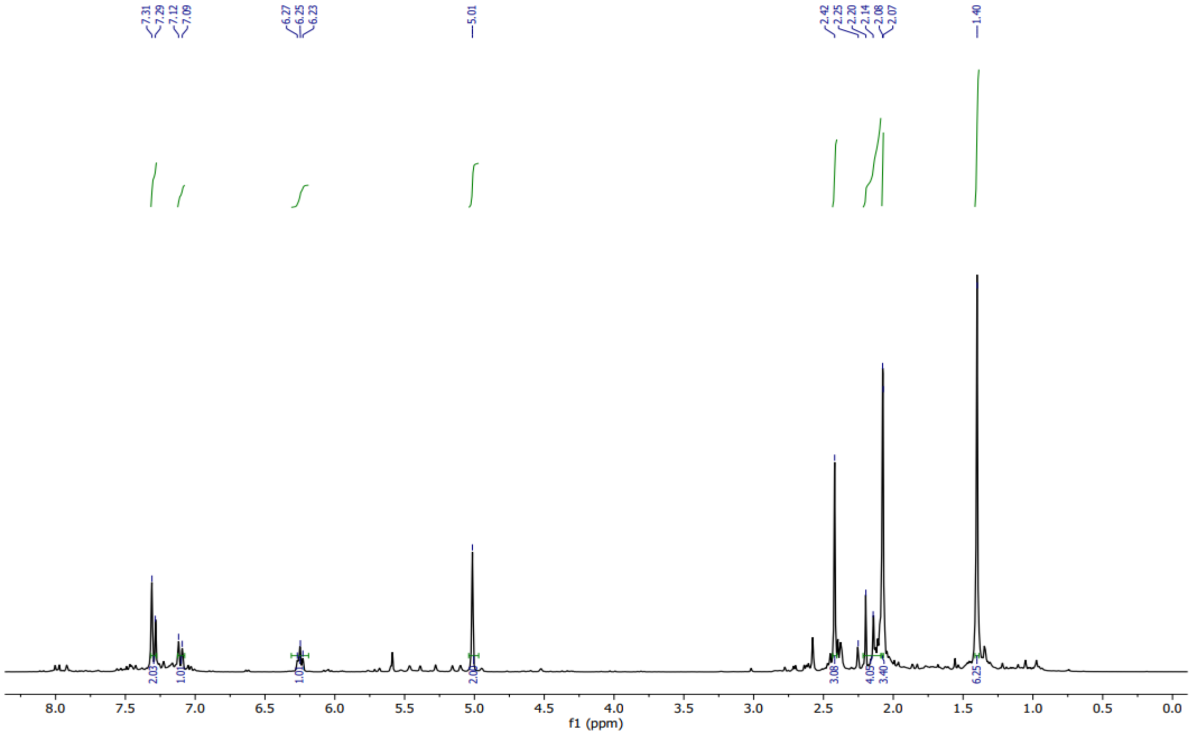

Figure S4. ^{1}H NMR Spectrum of **6**

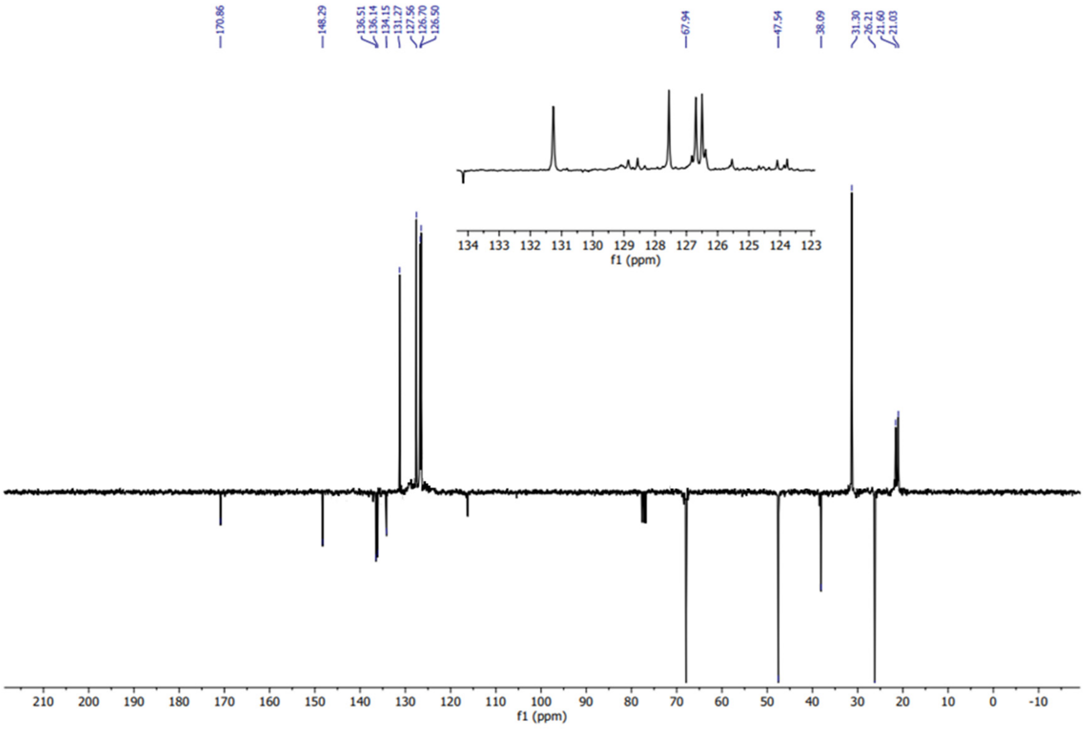

Figure S5. ^{13}C NMR Spectrum of **6**

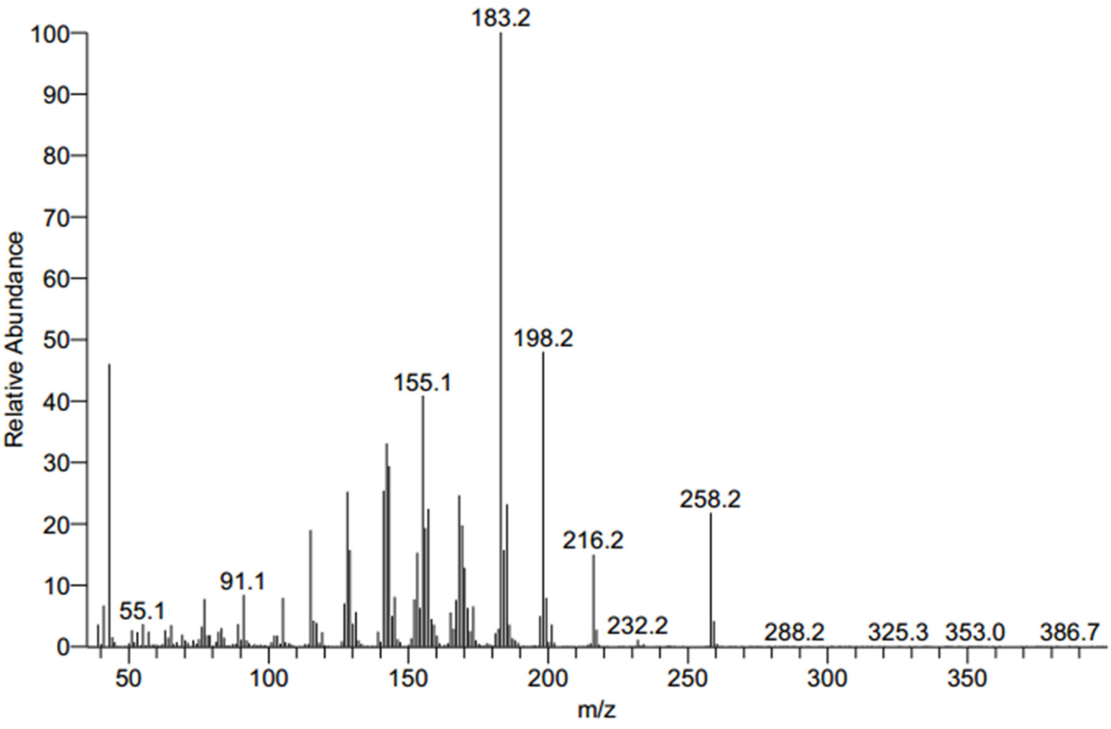

Figure S6. MS Spectrum of **6**

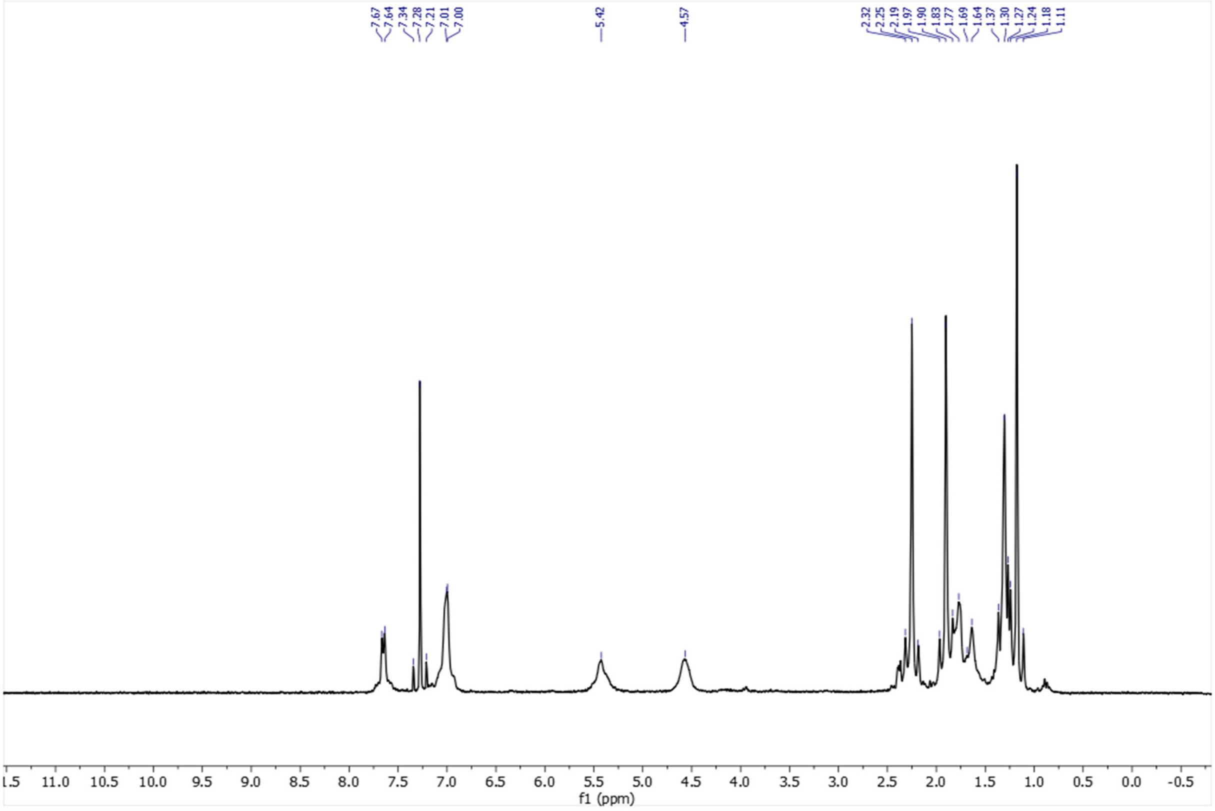

Figure S7. ^{1}H NMR Spectrum of **C1**

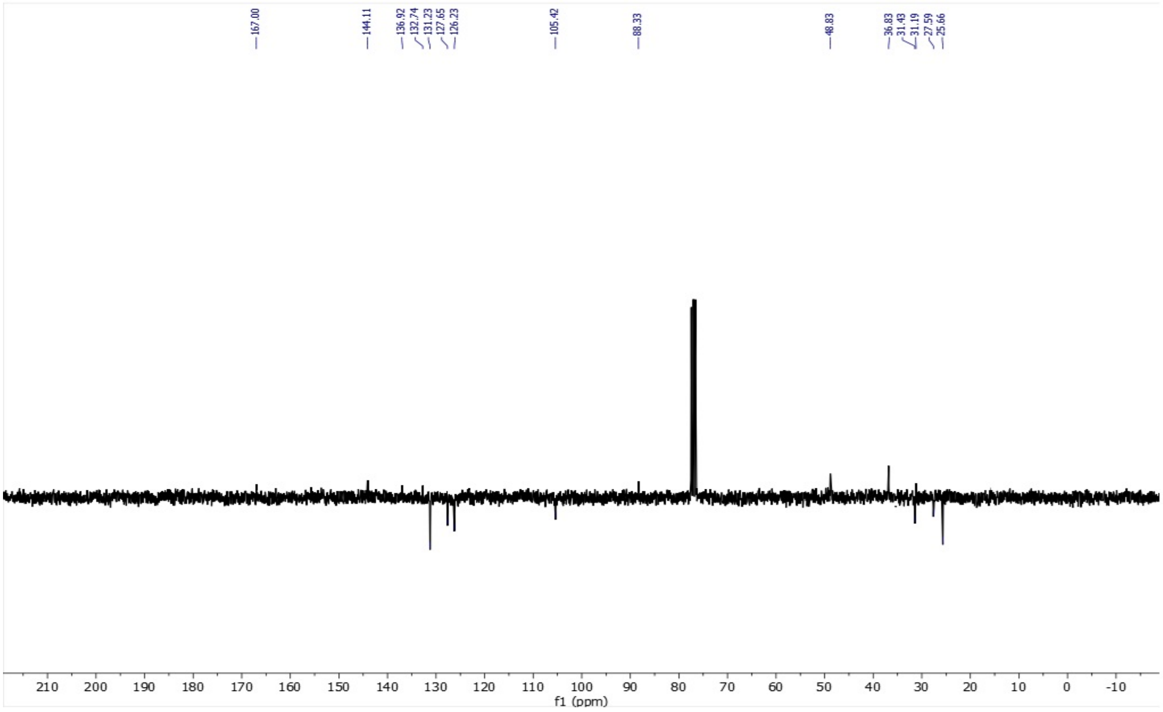

Figure S8. ^{13}C NMR Spectrum of **C1**
